# Tincture of Aconite Root and Veratrum Veride, in Puerperal Eclampsia

**Published:** 1880-11

**Authors:** 


TINCTURE OF ACONITE ROOT AND VERATRUM VERIDE, IN PUERPERAL ECLAMPSIA.—Dr. George M. Staples, of Dubuque, Iowa, reports several cases in which he used these articles hypodermically, two to three drops of the former and four to six of the latter, were the doses found necessary in arresting the spasms. The hypoderms were repeated at intervals of half to two hours, according to the urgency of the symptoms and duration of the attack.

